# Prevalence of Antimicrobial Resistance and Respective Genes among *Bacillus* spp., a Versatile Bio-Fungicide

**DOI:** 10.3390/ijerph192214997

**Published:** 2022-11-14

**Authors:** Pari Wash, Asiya Batool, Shah Mulk, Shabnum Nazir, Humaira Yasmin, Saqib Mumtaz, Mohammed Nasser Alyemeni, Prashant Kaushik, Muhammad Nadeem Hassan

**Affiliations:** 1 Department of Biosciences, COMSATS University Islamabad (CUI), Park Road, Islamabad 45550, Pakistan; 2 Botany and Microbiology Department, College of Science, King Saud University, Riyadh 11451, Saudi Arabia; 3 Instituto de Conservation y Mejora de la Agrodiversidad, Universitat Politecnica de Valencia, 46022 Valencia, Spain

**Keywords:** plant growth promoting rhizobacteria, *Bacillus* spp., multi drug resistance, MAR index

## Abstract

The plant rhizosphere is not only a reservoir of microbes but also a hub of antimicrobial resistance genes. Rhizospheric *Bacillus* spp. are the potential bio-inoculants with a versatile application in agriculture as bio-fertilizer and bio-fungicide. In the current study, the potential bio-control agent that is the *Bacillus* species (n = 7) was screened for the antimicrobial resistance pattern to assess their risk before registering them as a bio-inoculant. All of the *Bacillus* spp. were categorized as multi-drug-resistant (MDR), bacteria but none of them was either pan-drug-resistant (PDR) or extensive-drug-resistant (XDR). The multiple antimicrobial resistance (MAR) index of *Bacillus* spp. was higher than the critical value (0.2). The *Bacillus* spp. showed resistance to antimicrobial classes such as β lactam, macrolides, sulfonamides, tetracycline, aminoglycosides, and lincosamide. Various antimicrobial resistance genes, namely *VmiR*, *ImrB*, *tetL*, *mphK*, *ant-6*, *penp*, and *bla OXA*, associated with different mechanisms of resistance, were also detected in *Bacillus* spp. The *Bacillus* spp. also showed stress-tolerance traits such as ACC deaminase and EPS activity except the strains MAZ-117 and FZV-34, respectively. A significant correlation was observed between the PGPR and antimicrobial resistance, which shows that they may have adapted drug-resistance mechanisms to tolerate the environmental stress. These findings suggest that bio-fungicidal *Bacillus* spp. could be used very carefully on a commercial scale.

## 1. Introduction

The accumulations of antimicrobials and antimicrobial-resistance genes (ARGs) in the environment have led to the emergence of multidrug-resistant bacteria in the environment [1]. Utilization of the antimicrobial-contaminated animal manure, sewage sludge, and untreated industrial water leads to the development of resistance in the indigenous micro flora possessing beneficial traits in different fields such as crop production and bioremediation [2]. The frequent use of antimicrobials agents in livestock as feed additives is responsible for about 75% of the antimicrobials released into the environment [3]. Infections caused by antimicrobial-resistant bacteria cause the death of about 700,000 people every year [4].

The plant rhizosphere is not only a reservoir of microbes but also a hub of antimicrobial-resistance genes. The rhizospheric microbes could harbor antimicrobial genes due to their mutual interaction, especially between the pathogenic and beneficial bacteria. Rhizospheric *Bacillus* spp. are potential bio-inoculants with a versatile application in agriculture as bio-fertilizers and bio-fungicides. They secrete various metabolites that play an important role in soil fertility, nutrient recycling, plant growth, and productivity of crops, and they also help the plants in overcoming biotic and abiotic stress [5]. These plant-benefitting bacteria include species of *Bacillus*, *Pseudomonas*, *Burkholderia*, and *Azospirillum*, etc. The *Bacillus* spp. are one of the most well-characterized and widely used plant growth-promoting rhizobacteria (PGPR). There are numerous studies about the abundance of ARGs in agriculture soil, but less attention has been paid to the presence of drug-resistance traits in PGPR. The rhizospheric soil, enriched with ARGs, is the main source of the emergence and spread of antimicrobial resistance in the human food chain [6]. Antimicrobial resistance in bacteria can be intrinsic, acquired, and/or adaptive [7]. The intracellular cytoplasmic membrane is the major target of the most antimicrobial agents because of consequent changes in cell permeability and the presence of efflux pumps. These efflux pumps are present naturally in bacteria, and they are essential for bacterial survival and growth despite their exposure to various environmental challenges [8]. Various types of efflux pump families have been characterized in bacteria, such as the major facilitator superfamily, the small multidrug-resistance family, the resistance nodulation cell division family (RND), the ATP-binding cassette family, and the multidrug/toxic compound extrusion family [9]. Hydrophilic antibiotics, i.e., β lactam and tetracycline, cross the membrane barrier by water-filled channels known as porins. The alteration of porins in conjugation with the overexpression of efflux pumps leads to antimicrobial resistance. The Mef gene which is present in *Streptococcus* and other Gram-positive bacteria, is responsible for efflux-mediated resistance of macrolides. Other efflux pumps in Gram-positive bacteria include MsrA and MsrC, belonging to the ATP-binding cassette transporter family [10], while acquired resistance occurs through conjugation, transduction, and transformation. These include the alterations in enzymes involved in cell wall synthesis, nucleic acid synthesis, and protein synthesis, which include DNA gyrase, topoisomerase, and penicillin-binding protein [11]. The mobile genetic elements of bacteria, such as transposon, integrons, and plasmids, facilitate the horizontal transfer of ARGs among the soil bacteria [2]. Antimicrobial resistance could be a potential risk associated with the application of PGPR in soil, but it has been thus far ignored. Therefore, the present study aims to detect the antimicrobial-resistance pattern and respective AMRs genes in PGPR to be used as bio-fungicide.

## 2. Materials and Methods

### 2.1. Rhizobacteria and Culture Conditions

The strains of *Bacillus* spp. (n = 7) exhibiting bio-control activity against economically important pathogens of different crops (Table 1) were obtained from Applied Microbiology and Biotechnology Laboratory (AMBL), COMSATS University Islamabad (CUI), Pakistan. These strains were stored in 20% glycerol at −80 °C. The *Bacillus* spp. were grown on Luria Bertani (LB) agar (10 g L^−1^ trypton (Biolab, Budapest, Hungary), 5 g L^−1^ NaCl (Carl Roth, Karlsruhe, Germany), 5 g L^−1^ yeast extract (Biochem, Karlsruhe, Germany), and agar (Biochem, Karlsruhe, Germany) 10 g L^−1^) at 37 ± 2 °C [12].

### 2.2. Assessment of Drug-Resistance Determinents

#### 2.2.1. Phenotypic Assay

*Bacillus* spp. were tested for the susceptibility/resistance to different antimicrobial agents by the Kirby and Bauer’s disk diffusion method. The class and the number of antimicrobial agents for *Bacillus* spp. were selected by following the criteria of *Staphylococcus* given by clinical laboratory standards institute CLSI (Table 2) [19,20]. Each strain of *Bacillus* spp. was spread on LB agar to make a lawn and dried by keeping at room temperature for 2–5 min. The antimicrobial discs were placed on each agar plate by using automatic disc dispenser. The plates were incubated at 30 ± 2 °C for 24 h and were observed for the appearance of an inhibition zone around each colony. Based on inhibition zone diameter, the bacteria were categorized as resistant, susceptible, and intermediate by following the criteria of CLSI and/or EUCAST (European Committee on Antimicrobial Susceptibility Testing) [19,21].

#### 2.2.2. Multiple Antimicrobial Resistance Index (MAR Index)

The multiple antimicrobial resistance (MAR) index of the isolates was determined by the formula given below [22].

MAR index (multiple antimicrobial resistance) = Total number of antimicrobial resistance cases/Total number of antimicrobial agents used

### 2.3. Molecular Typing of Antimicrobial Resistance Genes of Bacillus spp.

#### 2.3.1. DNA Extraction and Primers

DNA was extracted by cetyltrimethylammonium bromide (CTAB) method [23]. Sixteen sets of primers (Table S1) targeting five major classes of ARGs were used to amplify the genes, namely aminoglycoside 3-N-acetyltransferase (*aac-3*), streptomycin 6-O-nucleotidyltransferase (*ant-6*), aminoglycoside 3′-O-phosphotransferase (*aph-3′*), macrolide 2’-phosphotransferase (*mphK*), lincomycin resistance (*lmrB*), (*VmiR*), penicillinase (*penp*), oxacillinase (*bla OXA*), subclass β1 metallo β-lactamase (*bla*), and tetracycline efflux MFS transporter (*tetL*) [24]. The DNA bands were electrophorized in 1 × TBE buffer using 1.2% (*w*/*v*) agarose gel pre-stained with ethidium bromide and visualized in a gel documentation system.

#### 2.3.2. PCR Amplification of AMR Genes

The drug-resistance genes were amplified through thermal cycler (peqSTAR, Deutschland, Germany) using cycling conditions given by [13] with slight modification including an initial denaturation at 94 °C for 5 min 45 s, with annealing temperature for each gene given in Table S1, and with final extinction at 72 °C for 11 min 30 s. A 25 μL reaction mixture was prepared by using PCR water (15.25 μL), 10 × Taq buffer (2.5 μL), 2 μL MgCl_2_ (25 mM), 0.5 μL dNTPs (10 mM), 1 μL of each primer (100 pM), 0.25 μL Taq polymerase (500 Units, Thermo Fisher Scientific, Waltham. MA, USA), and 2.5 μL template DNA (10–15 ng/μL). The previously amplified strain of *Bacillus* spp. (*B. subtilis* SM-23, *B. subtilis* SM-7, *B. velezensis* SM-22, and *B. halotolerans* SM-29) were used as a positive control, and DNA was replaced by distilled water in the negative control [24]. The amplified PCR product was analyzed in gel documentation system as described above.

### 2.4. Assessment of Stress-Tolerating Traits

#### 2.4.1. 1-Aminocyclopropane-1-Carboxylic Acid (ACC) Deaminase Activity

The *Bacillus* spp. were screened for the utilization of ACC as nitrogen source. *Bacillus* spp. were grown in LB medium at 37 °C with continuous shaking (120 rpm) for 24 h. A 24 h old culture of respective bacteria was inoculated at the center of the DF (Dworkin and Foster) medium supplemented with ACC [25]. Plates amended with ammonium sulphate were used as a positive control. Plates were allowed to incubate at 37 °C for 3 days. The growth of bacteria on ACC-supplemented plates was compared to both the positive and negative controls [26].

#### 2.4.2. Exopolysaccharide (EPS) Production

To determine the stress-tolerating ability of *Bacillus* spp., they were screened for EPS production on ATCC (American Type Culture Collection) no 4 medium as described by [27]. Bacterial colonies were streaked on ATCC no 4 medium and incubated at 37 °C for 3 days. Slime productions around the colonies were considered positive for EPS production.

### 2.5. Statistical Analysis

All tests for phenotypic assessment were conducted in triplicates at the least. The Pearson’s correlation was carried out between the genotypic and phenotype resistance of antimicrobial agents, ARGs, and PGPR traits. A correlation plot was constructed with OriginPro, version 2022, using “correlation plot” application. Additionally, a heat map showing prevalence of ARGs was compiled with the same tool using “split heat map” plot. A *p*-value greater than 0.05 was considered as statistically significant.

## 3. Results

### 3.1. Phenotypic Resistance and MAR Indices

**Figure 1:**
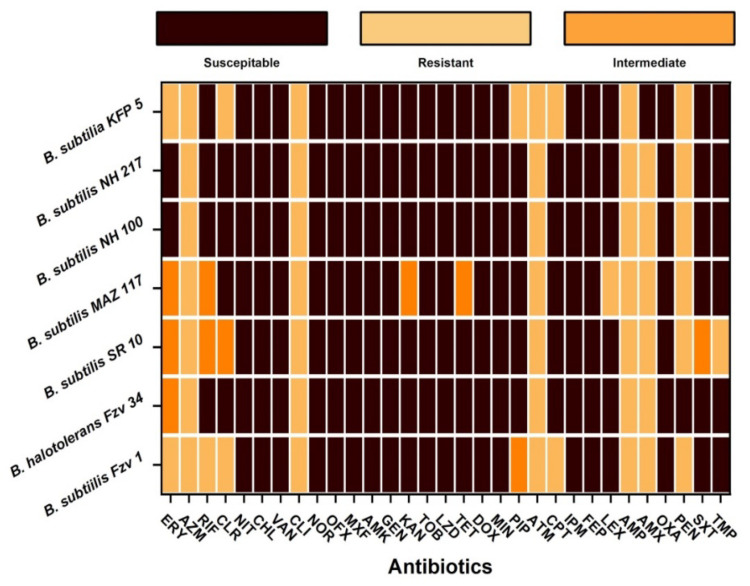
Prevalence of antimicrobial resistance in the *Bacillus* spp. ERY, Erythromycin; AZM, Azithromycin; RIF, Rifampin; CLR, Clarithromycin; NIT, Nitrofurantoin; CHL, Chloramphenicol; VAN, Vancomycin; NOR, Norfloxacin; OFX, Ofloxacin; MXF, Moxifloxacin; AMK, Amikacin; GEN, Gentamycin; TOB, Tobramycin; LZD, Linezolid; DOX, Doxycycline; MIN, Minocycline; PIP, Piperacillin; IPM, Imipenem; FEP, Cefepime; LEX, Cefalexin CLI, Clindamycin; OXA, Oxacillin; ATM, Aztreonam; CPT, Ceftaroline; AMP, Ampicillin; AML, Amoxicillin; PEN, Penicillin; SXT, Sulfamethoxazole; TMP, Trimethoprim; KAN, Kanamycin; TET, tetracycline.

The *Bacillus* spp. showed resistance to antimicrobial agents from different classes as shown in Figure 1 and Table S2. Antimicrobial susceptibility reveals that *Bacillus* spp. showed the highest resistance to β lactams, macrolides, and lincosamide (100%), followed by tetracycline (40%) (Figure 1 and Table S2). *Bacillus* spp. showed the least resistant to the sulfonamide (30%) and aminoglycoside (20%) classes of antimicrobial agents. Overall, all the tested *Bacillus* spp. showed resistance to azithromycin, clindamycin, aztreonam, and ampicillin (100%), followed by amoxicillin, penicillin, clarithromycin (85.7%), sulfamethoxazole, and rifampin (42.8%), while the least resistance was shown to trimethoprim (28.5%), cefalexin, kanamycin, and tetracycline (14.2%). All the tested strains were resistant to at least six antimicrobial agents, while one isolate showed resistance to ten antimicrobial discs. All the strains were characterized as multidrug-resistant (MDR) based on the resistance to at least three antimicrobials from different classes.

**Figure 2:**
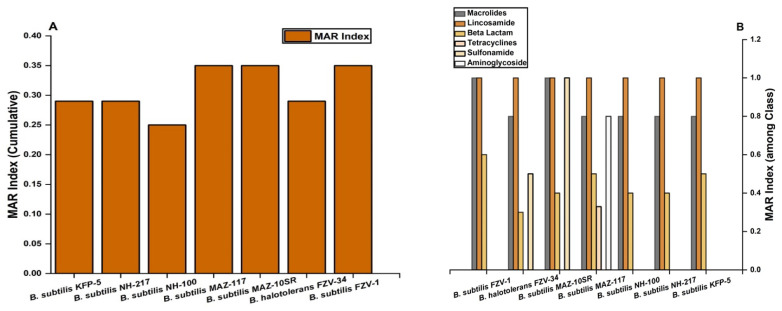
MAR indices of *Bacillus* spp.: (**A**) cumulative; (**B**) among class.

All the *Bacillus* spp. showed cumulative MAR indices greater than 0.2 as shown in Figure 2b. The highest MAR index (cumulative) was shown by *B. subtilis* MAZ-117, *B. subtilis* MAZ-10SR, and *B. subtilis* FZV-1 (MAR = 0.35), followed by *B. subtilis* KFP-5, *B. subtilis* NH-217, *B. halotolerans* FZV-34 (MAR = 0.29), and *B. subtilis* NH-100 (0.25). In case of different antimicrobial classes, the highest resistance was shown against macrolides (MAR = 0.8–1), lincosamide (MAR = 1), and β lactams (MAR = 0.3–0.6), followed by sulfonamide (MAR = 0.5–10, tetracycline (MAR = 0.33), and aminoglycoside (MAR = 0.8) as shown in Figure 2a. All the *Bacillus* spp. were susceptible to the nitrofurantoin, phenicols, glycopeptides, fluoroquanolones, and oxazolidinones classes of antimicrobials agents (MAR = 0) (Figure 1).

### 3.2. Detection of Antimicrobial-Resistance Genes in the Bacillus spp.

**Figure 3:**
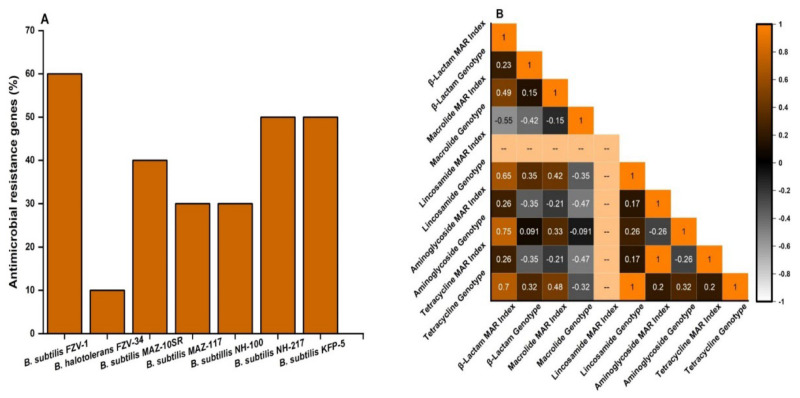
Presence of antibiotic-resistance genes in *Bacillus* spp. and correlation between resistant phenotype and genotype. (**A**) Frequency of antimicrobial resistance genes in *Bacillus* spp. (**B**) Phenotypic and genotypic correlation between antimicrobial resistance agents.

The number of genes encoding antimicrobial resistance (AMR) was found to be different in the *Bacillus* spp. as depicted in their resistance genotype (Table S2, Figure 3a). The maximum number of genes were detected in *B. subtilis* FZV-1 (60%), followed by *B. subtilis* KFP-5 and *B. subtilis* NH-217 (50%). The prevalence of antimicrobial resistance genes in *B. subtilis* MAZ-10 SR was 40%. In *B. subtilis* MAZ-117 and *B. subtilis* NH-100, the frequency of prevalence was 30; however, the antimicrobial-resistance genes were less prevalent in *B. halotolerans* FZV-34 (10%) as shown in Figure 3a. The most prevalent genotype was *VmiR* and *ImrB* (detected in six *Bacillus* spp.), followed by *tetL* (detected in five *Bacillus* spp.) and *mphK* (detected in four *Bacillus* spp.). The *penp* (detected in two *Bacillus* spp.), *ant-6* (detected in two *Bacillus* spp.), and *bla OXA* (detected in one *Bacillus* spp.) were the least-expressed genotype among *Bacillus* spp. The AMRs genes, namely *aac-3*, *aph-3*, and *bla2*, were not detected in any of the *Bacillus* spp. A close relationship was found between the βlactam MAR index and βlactam genotype (r = 0.23) and tetracycline MAR index and genotype (r = 0.2); however, the least correlation was found between macrolide MAR index and genotypes and aminoglycoside MAR index and genotypes (r = −0.15 and −0.26) as shown in Figure 3b.

### 3.3. ACC Deaminase and Exopolyscharride Production

**Figure 4:**
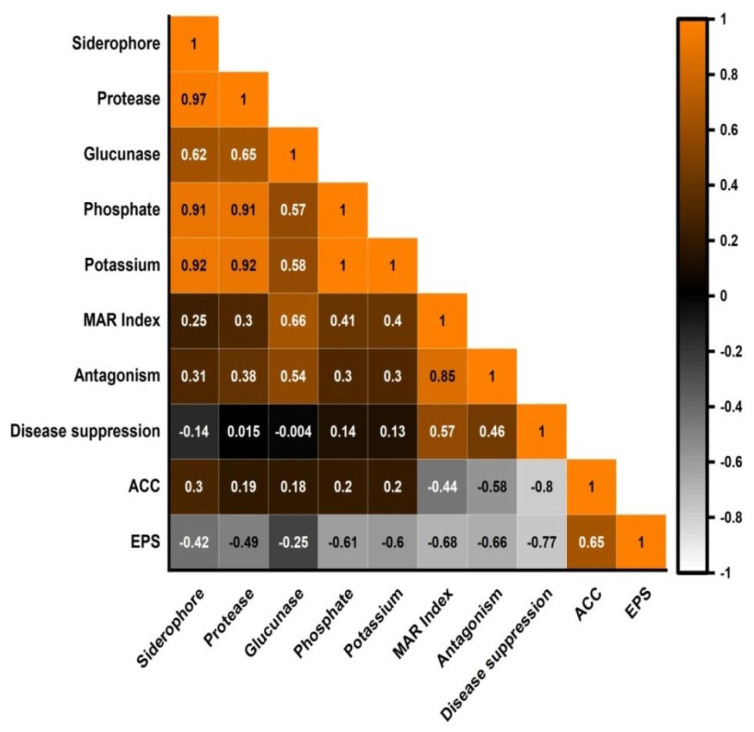
Correlation between MAR index and PGPR traits. ACC, 1-aminocyclopropane-1-carboxylic acid; EPS, exopolysaccharides; MAR index, multiple antimicrobial resistance index.

The *Bacillus* spp. grew well on DF medium supplemented with ACC except *B. subtilis* MAZ-117, which showed its ability to utilize ACC as a nitrogen source and exhibited ACC deaminase activity. All the species also produced exopolysaccharides except *B. halotolerans* FZV-34 (Table 3). The correlation plot (Figure 4) showed a strong correlation between MAR index and PGPR traits. The highest correlation was found between antagonism and MAR index (r = 0.85) followed by glucunase and MAR index (r = 0.66), disease severity and MAR index (r = 0.46), phosphate solubilization and MAR index (r = 0.41), potassium solubilization and MAR index (r = 0.4), protease and MAR index (r = 0.3), siderophore and MAR index (r = 0.25), ACC deaminase and MAR index, and EPS production and MAR index (r = 0.44, 0.68).

## 4. Discussion

The plant rhizosphere is an important reservoir of antimicrobial resistance determinants. PGPR inhibit the rhizosphere and could harbor antimicrobial-resistance traits. Their application at commercial scale may augment the antimicrobial resistance genes in rhizosphere. Moreover, these resistance determinants could disseminate to the clinical isolates through horizontal gene transfer [2]. Hence, characterization of drug-resistance determinants among PGPR is essential to utilize them as commercial bio-inoculants.

In the current study, based on the resistance pattern, all the *Bacillus* spp. were categorized as multidrug-resistant (MDR) bacteria because they resisted at least one antimicrobial disc among the three different classes of antimicrobial agents. No strain was found as XDR (Extensive Drug Resistance) and PDR (Pan Drug resistance) [28]. The resistance potential of multidrug-resistant bacteria is also expressed as MAR. The tested *Bacillus* spp. showed an MAR of 0.29–0.35, which was greater than the critical value of MAR, i.e., 0.2. A bacterium having MAR ≥ 0.2 is a potential hazard to the environment. The possible reason for higher MAR index in rhizobacteria could be due to the higher anthropogenic contamination and exchange of antimicrobial resistance genes among the beneficial and pathogenic bacteria inhabiting a common habitat such as a soil rhizosphere [29]. The resistance pattern against various antimicrobial classes was also found highly variable among the different strains. The highest phenotypic resistance was shown against the classes β lactam, macrolides, and lincosamide (100%), while a lower resistance was found against sulfonamide (28%), tetracycline, and aminoglycosides (14%). The *Bacillus* spp. were susceptible to nitrofurantoins, glycopeptides, oxazolidinones, phenicol, and fluoroquinolones. These findings are similar to the earlier reports by [28], where *Staphylococcus aureus* isolated from raw meat was resistant/susceptible to above-mentioned classes. The resistance/susceptibility to various antimicrobial classes by *Bacillus* spp. has already been reported in different studies [29,30,31]. However, for the first time, we report the categorization of plant-benefitting *Bacillus* spp. as MDR based on their resistance/susceptibility against eleven different classes. The resistance/susceptibility of bacteria to a particular class could be due to the adaption of specific mechanisms, such as modification of the cell wall permeability and change in the number of membrane transporter/porins and efflux pumps [32,33]. These mechanisms could be detected biochemically or genetically [30].

The resistance pattern of bacteria within each class was also variable depending on the antimicrobial agent. The frequency of resistance to antimicrobial agents among the tested strains was 0–100%. The variable resistance to various antimicrobial agents by *Bacillus* spp. isolated from different habitats has already been reported in previous studies [34,35]. The drug resistance/susceptibility depends upon different factors such as, exposure to a particular antibiotic termed as doze-dependent susceptible (S-DD), and growth-dependent bacteria antibiotic interaction, termed as drug indifferences [35]. This is why some drugs are considered to be most effective against Gram-positive bacteria, such as linezolid, as observed by LEADER (Linezolid Experience and Accurate Determination of Resistance), while some are considered as the “forgotten drugs” because of their less-common use, such as nitrofurantoins [36].

The phenotypic resistance of antimicrobial agents is related to the genotypic resistance. Genotypic resistance consists of certain genes that encode different mechanisms of resistance among different bacteria. In the current study, the *Bacillus* spp., which were phenotypically susceptible/resistant to antimicrobial classes, harbor certain genes that are associated with genetic mechanisms such as efflux pumps and inactivation of antimicrobial agents by production of certain enzymes such as β lactamases, macrolide phosphotransferase, and aminoglycosides nucleotidyltransferases. The most frequent genes occurring in the *Bacillus* spp. were *VmiR*, *ImrB*, and *tetL* encoding efflux pumps, followed by *mphK*, *penp*, *ant-6*, and *bla OXA*, which encode a genetic mechanism of enzymatic inactivation of antimicrobial agents/antibiotics. The prevalence of an efflux-pump-mediated resistance mechanism in *Bacillus* could be due to the natural occurrence of efflux pumps in bacteria, which they use for maintaining pH, establishment of proper charge, and uptake of nutrients.

Drug resistance might occur if the antimicrobial agent resembles their natural substrate, or the selection of substrate by the pump is low [8]. The absence of certain genes in drug-resistant *Bacillus* spp. might be because of the presence of some novel mechanisms of resistance, such as enzymatic modifications by aminoglycoside 5-phosphotransferase (aph 5) and methylation in 23S rRNA [37,38]. The phenotypic expression of a certain antimicrobial agents does not always depend upon the resistance genotype and vice versa; that is why the Pearson correlation depicted between resistance phenotypes and genotypes was heterologous. These findings suggest that the presence/absence of efflux pumps and their expressions should be detected biochemically.

Bio-fungicidal *Bacillus* spp. have been found to play an important role in the elevation of biotic and abiotic stress [5]. In the current study, the majority of the *Bacillus* spp. also showed stress-tolerance traits. The existence of PGPR, stress-tolerance, and drug-resistance traits shows that *Bacillus* spp. could use the same mechanism for both drug resistance and PGPR activities. A comprehensive understanding requires plasmid profiling and the locational analysis of the AMR genes in these strains to assess their impact on the environment.

## 5. Conclusions

The present study showed that bio-fungicidal *Bacillus* spp. carries drug-resistance traits and respective genes. Hence, they could be a source of transferring multidrug-resistance traits to other soil bacteria. This fact could limit their use in the environment. However, further investigation is required to explore the origin of drug resistance, respective mechanisms, and escape rate of the resistance determinants to assess the risk associated with their commercial use.

## Figures and Tables

**Table 1:** Plant growth-promotional and antagonistic properties of *Bacillus* spp.

Strains	16S rRNA ^a^ Accession No.	In Vitro PGPR ^b^ Traits					In Vivo PGPR ^b^ Activity	Antagonistic Activity (% ^c^)	References
		Sideropore (mm) ^d^	Proteases (mm) ^e^	Glucunase (mm) ^e^	Phosphate (mm) ^e^	Potassium (mm) ^e^	Disease Suppression (% ^c^)		
***B. subtilis*** ***MAZ-10SR***	MH598974	-	-	14.44	8.6	-	57	62.7	[13]
***B. subtilis*** ***MAZ-117***	MH702001	13.3	23.3	14.44	15	14.33	64.157	64.5	[14]
***B. subtilis*** ***KFP-5***	KT380825	7.2	15	11.9	2.8	3.0	44	63	[15,16]
***B. subtilis*** ***NH-100***	EU627167	-	-	-	3.2	-	56	53.3	[12,17]
***B. subtilis*** ***NH-217***	EU627170	-	-	-	5.2	-	53	60	[12,17]
***B. subtilis*** ***FZV-1***	MN810073	-	2.2	4.6	-	-	89	67.6	[18]
***B. halotolerans*** ***FZV-34***	MN810074	3.8	2.3	5.2	-	-	30	61.1	[18]

^a^ 16S rRNA, 16S small ribosomal subunit; ^b^ PGPR, plant growth-promoting rhizobacteria; ^c^ %, percentage; ^d^ discoloration zone in millimeters; ^e^ solubilization zone in millimeters.

**Table 2:** List of antimicrobial agents used in this study.

Sr. No.	Antimicrobial Name	Antimicrobial Class
1	Erythromycin (15 µg)	Macrolides
2	Azithromycin (15 µg)	
3	Rifampin (5 µg)	
4	Clarithromycin (15 µg)	
5	Nitrofurantoin (300 µg)	Nitrofurantoin
6	Chloramphenicol (30 µg)	Phenicol
7	Vancomycin (30 µg)	Glycopeptides
8	Clindamycin (2 µg)	Lincosamide
9	Norfloxacin (10 µg)	Fluoroquinolones
10	Ofloxacin (5 µg)	
11	Moxifloxacin (5 µg)	
12	Gentamicin (10 µg)	Aminoglycosides
13	Kanamycin (30 µg)	
14	Tobramycin (10 µg)	
15	Amikacin (30 µg)	
16	Linezolid (30 µg)	Oxazolidinones
17	Tetracycline (30 µg)	Tetracyclines
18	Doxycycline (30 µg)	
19	Minocycline (30 µg)	
20	Piperacillin (100 µg)	β lactam
21	Aztreonam (30 µg)	
22	Ceftaroline (30 µg)	
23	Imipenem (10 µg)	
24	Cefepime (30 µg)	
25	Cefalexin (30 µg)	
26	Ampicillin (10 µg)	
27	Amoxicillin (30 µg)	
28	Oxacillin (5 µg)	
29	Penicillin (10 µg)	
30	Sulfamethoxazole (25 µg)	Sulfonamide
31	Trimethoprim (5 µg)	

**Table 3:** ACC deaminase activity and EPS production in *Bacillus* spp.

Isolates	ACC Deaminase Activity	EPS Production
*B. subtilis MAZ-117*	−	+
*B. subtilis* *-10SR*	+	+
*B. subtilis* *KFP-5*	+	+
*B. subtilis* *NH-100*	+	+
*B. subtilis NH-217*	+	+
*B. subtilis FZV-1*	+	+
*B. halotolerans* *FZV-34*	+	−

“+” indicates positive; “−” indicates negative.

## Data Availability

Not applicable.

## Supplementary Materials

The following supporting information can be downloaded at: https://www.mdpi.com/article/10.3390/ijerph192214997/s1, Table S1: List of Primers, their annealing temperatures, and amplicon size; Table S2: Resistance genotype and phenotype of Bacillus spp.
