# Supplementary material for: Prevalence of Antimicrobial Resistance and Respective Genes among Bacillus spp., a Versatile Bio-Fungicide
Source: Int J Environ Res Public Health. 2022 Nov 14;19(22):14997. doi: 10.3390/ijerph192214997 (PMC9690011; doi:10.3390/ijerph192214997)
Supplement: Supplementary file 1 [file ijerph-19-14997-s001.zip › ijerph-1995405-supplementary.pdf]

Supplementary Material

Table S1. List of primers, their annealing temperature and amplicon size.

Genes	Primers	Sequence (5'-3')	Tm (°C) Net Primer	Amplicon (bp)
penp	penp-ST-F	TTGTTACATATTCTCCAATCACAG	55	455
	penp-ST-R	GGCCAAACGATAGCAATG	55	
blaOXA	blaOXA-S-F	TGGGACGGGGTCAAGCG	63	300
	blaOXA-S-R	TCTTGAATCATCATGCGCTTGAC	63	
vmiR	vmlR-S-F	GAAGCGTACGGATGCCC	58	798
	vmlR-S-R	CCAGCACTTCTTGTCTTTCTG	57	
ImrB	lmrB-S-F	GATGCCGATTATGATTTCCTTG	59	850
	lmrB-S-R	ATCGGCAGAAGCAGCATAG	56	
aac-3	aac-3-S-F	GGGATGACCGTACTTGTACATTCCT	63	638
	aac-3-S-R	ACTTTGCCAACTTTCATATTATGTTCT	60	
ant-6	ant-6-H-F	TGGTTACTTTGGAAGGATCACG	59	655
	ant-6-H-R	TTCCACATTTCTTGATACCCATTC	60	
ant-6	ant-6-S-F	TGGACTTTGCTTTGAACGATGAG	62	799
	ant-6-S-R	CCTTCCGTATACTTAGTGATGCCTTC	62	
mphK	mph-2-SH-F	AATCGGGATGAATCTGCCTATGTA	62	1181 (H)<br>1181 (S)
	mph-2-SH-R	CACCTGCCACCGACTCTCTGA	63	
tetL	tet-S-F	GCGTATCAATGACCCTATAAACTG	58	1401
	tet-S-R	CATTCAAGATTACTAGCCAACAAATAA	59	

blaOXA (oxacillinase), *penp* (penicillinase), *bla* (subclass B1 metallo β-lactamase), *lmrB* (lincomycin resistance), *aac-3* (aminoglycoside 3-N-acetyltransferases), *ant-6* (streptomycin 6-O-nucleotidyl transferase), *aph-3′* (aminoglycoside 3′-O-phosphotransferases), *mphK* (macrolide 2'-phosphotransferase), and *tetL* (tetracycline efflux MFS transporter).

Table S2. Resistance genotype and phenotype of *Bacillus* spp.

Strains	Resistance phenotype	Antibiotic Class	Resistance genotype	Mechanism
B.subtilis MA117	ERY, AZM, RIF, CLR,	Macrolide	N/A	
	CFX, AML, PEN, ATM, AMP	B lactam	N/A	
	KAN	Aminoglycoside	N/A	
	TET	Tetracycline	*tetL*	Efflux pump
	CLI	Lincosamide	*VmiR*, *ImrB*	Efflux pump
B. subtilis 10SR	ERY, AZM, RIF, CLR,	Macrolide	*mphK*	Inactivation by macrolide phosphotransferases
	CLI,	Lincosamide	*VmiR*, *ImrB*	Ribosomal protection, ABC efflux pump
	ND	Tetracycline	*tet L*	Efflux pump
	ATM, AMP, AML, PEN,	B lactam	N/A	
	SXT, TMP	Sulfonamide	ND	
B. subtilis KFP 5	ERY,AZM, CLR	Macrolide	*mphK*	Inactivation by macrolide phosphotransferases
	PRL, ATM, CPT, AMP, PEN	B lactam	N/A	
	CLI	Lincosamide	*VmiR*, *ImrB*	Ribosomal protection, ABC efflux pump
	ND	Tetracycline	*tetL*	Efflux pump
	ND	Aminoglycoside	*ant-6*	Inactivation by aminoglycosides nucleotidyltransferases
B. subtilis NH 100	ERY, AZM, CLR,	Macrolide	N/A	
	ATM, AMP, AML, PEN	B lactam	*penp*	Inactivation by Beta lactmases
	CLI	Lincosamide	*VmiR*, *ImrB*	Ribosomal protection, Efflux pump
B. subtilis NH 217	ERY, AZM	Macrolide	*mphK*	Inactivation by macrolide phosphotransferases

	ATM, AMP, AML, PEN,	B lactam	*bla OXA*	Inactivation by beta lactmases
	SXT	Sulfonamide	ND	
	CLI	Lincosamide	*VmiR*, *ImrB*	Efflux pump
	ND	Tetracycline	*tet L*	Efflux pump
B. subtilis FZV 1	ERY, AZM, CLR, RIF,	Macrolide	*VmiR* , *ImrB*	Efflux pump
	CLI,	Lincosamide	ND	
	PRL, ATM, CPT, AMP, AML, PEN	B lactam	*penp*	Inactivation by beta lactmases
	ND		*ant-6*	Inactivation by aminoglycosides nucleotidyltransferases
	ND		*tet L*	Efflux pump
B. halotolerans FZV 34	ERY, AZM, CLR,	Macrolide	*mphK*	Inactivation by macrolide phosphotransferases
	ATM, AMP, AML,	B lactam	ND	
	SXT, TMP	Sulfonamide	ND	
	CLI	Lincosamide	ND	

ERY = Erythromycin; AZM = Azithromycin; RIF = Rifampin;CFX = Cefalexine; AML = Amoxicillin; PEN = Penicillin; KAN = Kanamycin; TET = Tetracycline; CLR = Clarithromycin; CLI = Clindamycin; ATM = Aztronam; AMP = Ampicillin; SXT= Trimethoprim sulphamethaxazole; TMP= Trimethoprim; PRL= Piperacillin; CPT= Ceftaroline
ND = Not Detected; N/A = Not applicable
